# Huayu Tongmai Granules protects against vascular endothelial dysfunction via up-regulating miR-185 and down-regulating RAGE

**DOI:** 10.1042/BSR20180674

**Published:** 2018-11-30

**Authors:** Xiaoming Liu, Dongli Wang, Xiaoni Yang, Lei Lei

**Affiliations:** 1 Doctor Candidates of 2014 Grade, Shandong University of Traditional Chinese Medicine, Jinan 250355, China; 2 Department of Geriatric, Affiliated Hospital of Shandong University of Traditional Chinese Medicine, Jinan 250014, China; 3 Department of Traditional Chinese Medicine, Shandong Qianfoshan Hospital, Jinan 250014, China; 4 Department of Special Examination, Shandong Qianfoshan Hospital, Jinan 250014, China

**Keywords:** Chinese medicine, diabetic angiopathy, endothelial cells, miR-185, RAGE

## Abstract

**Objective**: Receptor of advanced glycation end products (RAGE) is a membrane protein that contributes to the initiation and progression of diabetic vascular complications, which is reported as a target of miR-185. Huayu Tongmai Granules is a Chinese herbal compound that is capable of treating diabetic angiopathy. The present study was designed to explore the molecular biological mechanism by which Huayu Tongmai Granules protects against diabetic angiopathy.

**Methods:** The rat model of diabetes and hyperglucose cell model were established. The blood glucose was detected to verify whether the model was successfully established. Besides, serum nitric oxide (NO) and reactive oxygen species (ROS) concentrations of the rats in each group were determined. The quantitative real-time PCR analysis was performed to examine the mRNA expression levels of miR-185 and other miRNAs in femoral artery of rats or human umbilical vein endothelial cell line. Additionally, the protein levels of RAGE or Bax were determined using Western blotting. Cell apoptosis was determined by terminal dUTP nick-end labeling assay or flow cytometry.

**Results:** In the present study, we found that Huayu Tongmai Granules significantly decreased blood glucose and serum ROS and up-regulated serum NO concentration. MiR-185 was low-expressed in diabetic rats; however, Huayu Tongmai Granules intervention up-regulated miR-185. Stable overexpression of miR-185 directly suppressed the expression of RAGE and further suppressed endothelial cell apoptosis.

**Conclusion**: Huayu Tongmai Granules appears to have a therapeutic effect on diabetic angiopathy that is most probably mediated by miR-185/RAGE axis.

## Introduction

Diabetes mellitus (DM) is a metabolic disorder referring to a disorder in the metabolism of carbohydrates, fats and proteins, which is defined by hyperglycemia, affecting over 400 million patients worldwide [1]. Recently, accumulating evidence has strongly implied that diabetes has devastating effects on the vasculature leading to numerous vascular complications including cardiovascular diseases, retinopathy, neuropathy and nephropathy, which are also the major causes of morbidity and mortality [2]. There is a growing evidence supporting that oxidative stress, collagen deposition, angiogenesis, and vascular remodeling are associated with the pathogenesis of diabetic vascular complications [3–5]. Emerging evidence has confirmed that diabetic macroangiopathy is characterized by vascular endothelial dysfunction and vascular fibrosis and cirrhosis [6]. On the one hand, endothelial dysfunction results from impairment of vasodilation factor-nitric oxide (NO) synthesis and the apoptosis of endothelial cells. Moreover, fibrosis process consists of the promotion of extracellular matrix (ECM) production and advanced glycation end products (AGE)-dependent cross-linking of ECM [7].

Therefore, the extensive intracellular and extracellular formation of AGE is considered to be a causative factor in sustained hyperglycemia-induced diabetic angiopathy. Receptor of advanced glycation end products (RAGE) is a multiligand member of the immunoglobulin superfamily of cell surface molecules and now recognized as a pro-inflammatory molecular device mediating danger signals to the body, which interacts with a diverse class of ligands, including AGEs, amphoterin and amyloid-β peptide, since it was originally found as a signal transduction receptor for AGEs [8]. Additionally, it has been reported that AGEs bind to RAGE to induce oxidative stress, inflammation and apoptosis, which lead to endothelium damage [9]. Thus, exploring effective drugs to intervene AGEs-induced endothelial dysfunction has dramatic significance in the prevention and treatment of diabetic vascular complications.

MiRNAs are a class of small single-stranded non-coding RNAs containing approximately 21–23 nucleotides, which play crucial roles in physiological and pathological processes by regulating expressions of multiple proteins via directly binding to 3’UTR of target RNAs. Although many miRNAs have already been identified, their roles in the regulation of key genes and signaling pathways associated with diabetes pathology still remain largely unknown. Among several diabetes-related miRNAs, miR-185 has been recently identified to have specific translational inhibitory effect on the 3′-UTR of RAGE through direct interaction, involving in the development of esophageal squamous cell carcinoma [10, 11]. Hence, we speculated that the down-regulated miR-185 in diabetic patients and mice was involved in the progression of diabetic angiopathy via directly targeting RAGE.

The objective of the present study was to determine the underlying effects of a Chinese herbal compound called Huayu Tongmai Granules on the development of diabetic angiopathy. Our preliminary study has found that it could markedly improve the clinical symptoms of patients with diabetes, and also has the function to reduce serum sugar and lipid. Therefore, our study may elucidate the potential molecular mechanisms by which the compound protects against vascular endothelial dysfunction in diabetes as based on traditional Chinese medicine (TCM) theories.

## Materials and methods

### Induction of diabetes and experimental design

Three-month-old male Sprague–Dawley rats with 300–330 g of body weight purchased from Shandong University of Traditional Chinese Medicine (Jinan, Shandong, China) were housed individually in an air-conditioned room maintained at a temperature of 22°C ± 2°C and 12/12-h light/dark cycle and were fed a normal rodent diet or high-fat, carbohydrate-free diet containing 72% fat (corn oil and lard), 28% protein, and 1% carbohydrate. After adaptive feeding for 1 week, the rats were randomly divided into four groups (*n*=6 per group) as follows: control, normal feeding rats, DM, and DM + NS. DM model rats were fed with high-fat and high-fructose diet as is described previously [12], including 4.1 kcal/g, 60% fructose, 10% lard, 20.7% casein, 4.2% fiber, 3.5% minerals, 1% vitamins, 0.3% calcium carbonate, and 0.3% dl-methionine. The DM rats were induced by intraperitoneal injecting of 30 mg/kg Streptozocin (STZ, Sigma), which was dissolved in the sterile citrate buffer (W/V 2%) [13]. The normal feeding rats were fed with 3.15 kcal/g, 5% fat, 21% protein, 60% starch, 3% fiber, and 1% vitamins and minerals. DM rats supplemented with 3 mg/kg normal saline; DM + HYTM, DM rats supplemented with 3 mg/kg Huayu Tongmai Granules. After 12 weeks, the peripheral blood glucose level was measured using the Precision Xtra Blood Glucose Monitoring System (Alameda, CA, U.S.A.). The model was successfully established if the blood glucose of each rat was over 16.7 mmol/l. Besides, serum NO and reactive oxygen species (ROS) concentrations of the rats in each group were determined. All animal experiments were performed in accordance with the Guidelines for Laboratory Animal Care and Use of Shandong University of Traditional Chinese Medicine. The femoral artery was separated from each group to detect the mRNA expressions of a series of miRNAs including miR-185, miR-92a, miR-712, miR-342-5p, and miR-320a by quantitative real-time PCR (qRT-PCR) and the protein levels of RAGE and Bax using Western blotting. In addition, endothelial cell apoptosis was determined by performing terminal dUTP nick-end labeling (TUNEL) assay.

### Cell line and cell treatment

Human umbilical vein endothelial cells (HUVECs) line was purchased from American type culture collection (ATCC) and cultured in low-glucose DMEM (Gibco, Carlsbad, CA, U.S.A.) supplemented with 10% FBS (Gibco) and 80 units/ml of penicillin/streptomycin (Gibco) at 37°C in a 95% air/5% CO_2_ atmosphere. To explore whether miR-185 could regulate vascular endothelial cell apoptosis, the cells were randomized to control, 25 mM, 25 mM + pre-NC, and 25 mM + miR-185 mimic groups. Briefly, HUVECs were maintained in low-glucose DMEM or high-glucose DMEM containing 25 mM glucose and 10% FBS for 20 h and transfected with pre-NC or miR-185 mimic. After 48 h, the cells were harvested. The miR-185 and Bax expression levels and cell apoptosis were determined by performing qRT-PCR, Western blotting, and flow cytometry, respectively.

### Cell transfection

To further understand the biological function and molecular roles of miR-185 in vascular endothelial cells, the inhibitors and the mimics of miR-185, pre-NC and pcDNA-RAGE plasmid or pcDNA vector were all designed by Shanghai GenePharma (Shanghai, China) and transfected into HUVECs cells by Lipofectamine 2000 (Invitrogen, Carlsbad, CA, U.S.A.), according to the manufacturer’s instructions, after HUVECs were cultured in low-glucose or high-glucose DMEM for 20 h. At 48 h after the transfection, the cells were harvested and were used for determining RAGE and Bax expressions at protein levels and cell apoptosis.

### HYTM treatment *in vitro*

The ingredients of HYTM were included in *Codonopsis pilosula*, the root of red-rooted salvia (*Salvia miltiorrhiza*), *Coptis chinensis, Ligusticum wallichii*, hawthorn, *Angelica sinensis, Carthamus tinctorious*, and Radix Paeoniae Alba.

We eventually investigated the actual effects of Huayu Tongmai Granules in vascular endothelial cells *in vitro*. HUVECs were cultured in high-glucose DMEM for 20 h, pretreated with 1 mg/ml HYTM for 24 h, and then transfected with NC or miR-185 inhibitor for 48 h. HUVECs cultured in high-glucose DMEM for 20 h were used as the control group. Cells were collected for miR-185, RAGE, Bax expression levels, and apoptosis testing.

### Determination of serum markers of diabetes

The detection of serum NO level was detected by using the Nitric Oxide Colorimetric Assay Kit (Nanjing Jiancheng Bioengineering Institute, Nanjing, Jiangsu, China) following manufacturer’s instruction. The ROS production was measured after the addition of 5 µl luminol (0.1 mM) working solution prepared in DMSO (Sigma–Aldrich, St. Louis, MO, U.S.A.) and 2 µl fMLP working solution (0.2 µM). The ROS value was expressed as the relative light units per min when the chemiluminescence signal was monitored for 15 min.

### RNA isolation and qRT-PCR

Total RNA was extracted by using Trizol reagent (Thermo Fisher Scientific, Waltham, MA, U.S.A.) according to the manufacturer’s instructions. The RNA samples were reverse transcribed using PrimeScript RT Master Mix (TaKaRa, Tokyo, Japan) after RNA isolation. The products were then subjected to real-time qPCR analysis using 7500 FAST Real-Time PCR System (Applied Biosystems, Carlsbad, CA, U.S.A.). All reactions were performed in triplicate. U6 snRNA was used as the internal control for miR-185 and other genes. The data were analyzed by using the the 2^−ΔΔ*C*^_t_ method.

### Western blot

Total protein was obtained from femoral artery and HUVECs using Radio-Immunoprecipitation Assay Buffer (Beyotime, Shanghai, China). Then, SDS lysis buffer (Beyotime) was added, and protein extracts were heated at 100°C for 5 min. Proteins were separated with 10% PAGE and then transferred onto a PVDF membrane. The membrane was blocked with tris-buffered saline tween (TBST) containing 5% skimmed milk for 2 h at room temperature and incubated with primary antibodies against RAGE (1:1000; Cell Signaling Technology, Boston, MA, U.S.A.) and Bax (1:1000; Cell Signaling Technology) at 4°C overnight. β-Actin monoclonal antibody was used as an internal reference. The membrane was washed with TBST and incubated with the peroxidase-labeled anti-rabbit secondary antibody (1:2000; Santa Cruz Biotechnology, Inc., Santa Cruz, CA, U.S.A.) for 1 h at room temperature and visualized using Chemiluminescent Reagent (Thermo Fisher Scientific).

### TUNEL assay

Paraffin section of rats’ femoral artery was taken to receive conventional dewaxing, antigen repair treatment, and blocked with sealing liquid. Sections were incubated and repaired for 15 min in the mixture working solution (20 μg/ml proteinase K dissolving in 10 mmol/l Tris-HCl, pH: 7.4–8; Sigma–Aldrich) at 37°C. A total of 50 μl TUNEL reaction solutions were added for 1-h incubation at 37°C, and then the sections were washed with PBST, incubated with diaminobenzidine for 30 min in converter-peroxidase at 37°C, and washed again with PBST. Samples were observed under the microscope (Olympus, Tokyo, Japan). The TUNEL Kit used in the experiment was purchased from Roche Applied Science (Mannheim, Germany). Four fields of each tissue were randomly selected for calculation, and the average value was collected.

### Flow cytometry

The apoptosis of HUVECs was analyzed by flow cytometry using Annexin V-FITC Apoptosis Detection Kit (BD Biosciences; San Jose, CA, U.S.A.) according to the manufacturer’s protocols. After being harvested and washed with cold PBS, cells were stained with binding buffer containing Annexin V-FITC and propidine iodide at 4°C under darkness for 15 min. Finally, cells were recorded using flow cytometry (Beckman Coulter, Fullerton, CA, U.S.A.).

### Statistical analysis

The data were expressed as mean ± S.D. Statistical analyses were performed using SPSS version 22.0 (SPSS, Chicago, IL, U.S.A.). Student’s *t*test was performed to compare the difference (*P*<0.05) between two groups. One-way ANOVA followed by the Newman–Keuls *post hoc* test was used to evaluate the statistical significance (*P*<0.05) between different groups. *P*<0.05 was considered to be statistically significant.

## Results

### Huayu Tongmai Granules significantly regulated serum markers of diabetes

**Figure 1:**
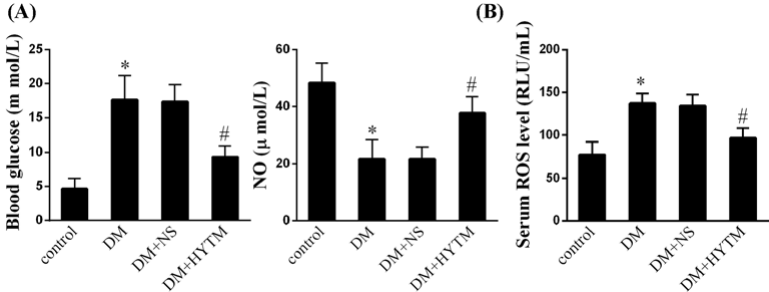
Effect of Huayu Tongmai Granules on serum markers of diabetes (**A**) Blood glucose and serum NO concentration in the groups of control, normal feeding rats; DM, DM model rats; DM + NS, DM rats supplemented with 3 mg/kg normal saline; DM + HYTM, DM rats supplemented with 3 mg/kg Huayu Tongmai Granules. (**B**) Serum ROS levels in the groups of control, DM, DM + NS, and DM + HYTM. Data were shown as mean ± S.D. *Compared with control group, *P*<0.05; ^#^compared with DM + NS group, *P*<0.05.

Compared with the control rats, blood glucose levels in DM rats significantly increased, accompanied by lower serum NO concentration (Figure 1A, *P*<0.05) and higher serum ROS levels (Figure 1B, *P*<0.05). Twelve-week Huayu Tongmai Granules administration significantly decreased the blood glucose levels accompanied by improvement of serum NO (Figure 1A, *P*<0.05) and reduction in ROS levels (Figure 1B, *P*<0.05). The results further verified anti-diabetic angiopathy effects of Huayu Tongmai Granules.

### Huayu Tongmai Granules up-regulated miR-185 in a rat model of diabetes

**Figure 2:**
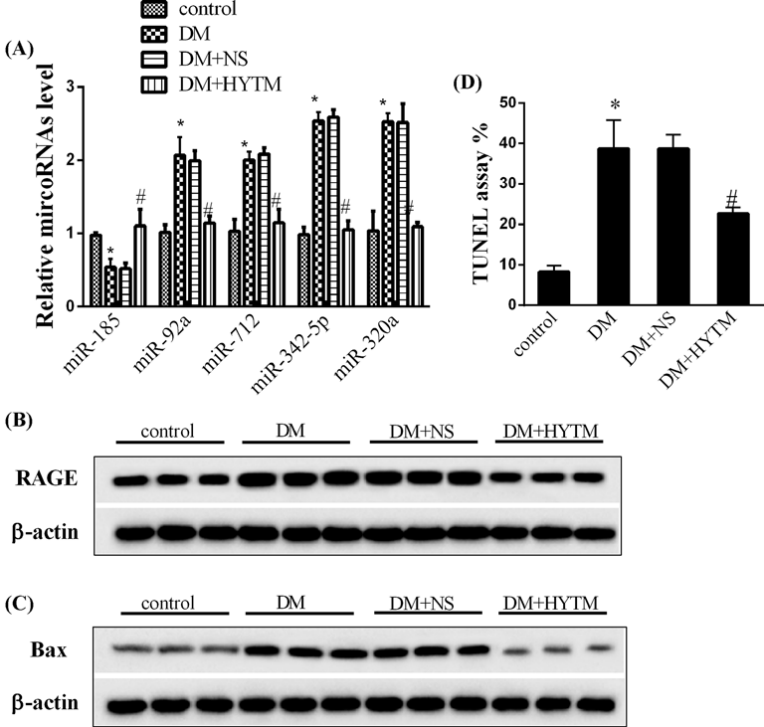
Effect of Huayu Tongmai Granules on gene expressions and endothelial cell apoptosis (**A**) The expression levels of several miRNAs in femoral artery in the groups of control, DM, DM + NS, and DM + HYTM. (**B**) The protein expression levels of RAGE in femoral artery in the groups of control, DM, DM + NS, and DM + HYTM. (**C**) The protein expression levels of Bax in femoral artery in the groups of control, DM, DM + NS, and DM + HYTM. (**D**) Cell apoptosis results in the groups of control, DM, DM + NS, and DM + HYTM. Data were shown as mean ± S.D. *Compared with control group, *P*<0.05; ^#^compared with DM + NS group, *P*<0.05.

To identify the potential miRNAs that may be involved in the progression of diabetic angiopathy, we chose five previously reported lncRNAs and subjected them to qRT-PCR analysis to determine their expression during DM: miR-185, miR-92a, miR-712, miR-342-5p, and miR-320a. As shown in Figure 2A, four miRNAs, including miR-92a, miR-712, miR-342-5p and miR-320a, were elevated in DM rats and were decreased by Huayu Tongmai Granules treatment (*P*<0.05). Especially, miR-185 exhibited marked down-regulation in DM, and up-regulated after Huayu Tongmai Granules administration (*P*<0.05). RAGE and the apoptosis-effector gene, Bax, were also examined and they displayed significant increase in DM rats, but exhibited marked down-regulation by Tongmai Granules administration (Figure 2B,C, all *P*<0.05). The results of TUNEL assay also supported this fact that DM promoted the apoptosis of endothelial cells, while Tongmai Granules inhibited the apoptosis under high-glucose condition (Figure 2D, *P*<0.05). In addition, cell apoptosis under the treatment of insulin was also explored. The results revealed that insulin stimulation did not affect cell apoptosis (Supplementary Figure S1, *P*<0.05).

### MiR-185 overexpression inhibited endothelial cell apoptosis

**Figure 3:**
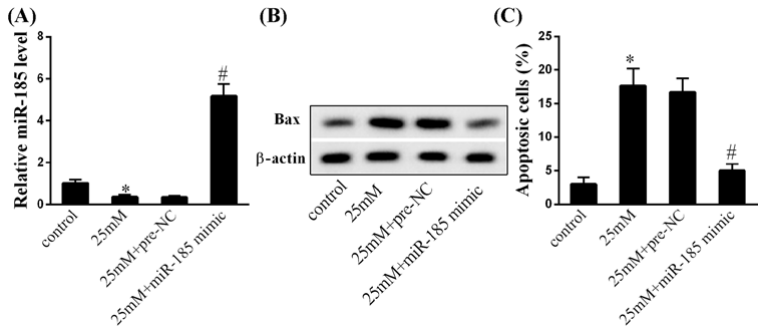
Effect of miR-185 overexpression on endothelial cell apoptosis (**A**) The expression levels of miR-185 in HUVECs in the groups of control, cultured in low-glucose DMEM; 25 mM, cultured in high-glucose DMEM containing 25 mM glucose; 25 mM + pre-NC, cultured in high-glucose DMEM containing 25 mM glucose and transfected with pre-NC; 25 mM + miR-185 mimic, cultured in high-glucose DMEM containing 25 mM glucose and transfected with miR-185 mimic. (**B**) The protein expression levels of Bax in HUVECs in the groups of control, 25 mM, 25 mM + pre-NC, and 25 mM + miR-185 mimic. (**C**) The percentage of apoptotic HUVECs in the groups of control, 25 mM, 25 mM + pre-NC, and 25 mM + miR-185 mimic. Data were shown as mean ± S.D. *Compared with control group, *P*<0.05; ^#^compared with 25 mM + pre-NC group, *P*<0.05.

We then evaluated the biological effect of miR-185 on endothelial cell apoptosis. Pretreatment of high-glucose DMEM significantly down-regulated miR-185 mRNA expression (Figure 3A, *P*<0.05), up-regulated Bax protein levels (Figure 3B, *P*<0.05) in HUVECs and induced the apoptosis of HUVECs (Figure 3C, *P*<0.05), which were reversed by miR-185 overexpression. These findings indicated that miR-185 overexpression might exert anti-apoptosis effect on endothelial cells.

### MiR-185 directly targeted RAGE to inhibit endothelial cell apoptosis

**Figure 4:**
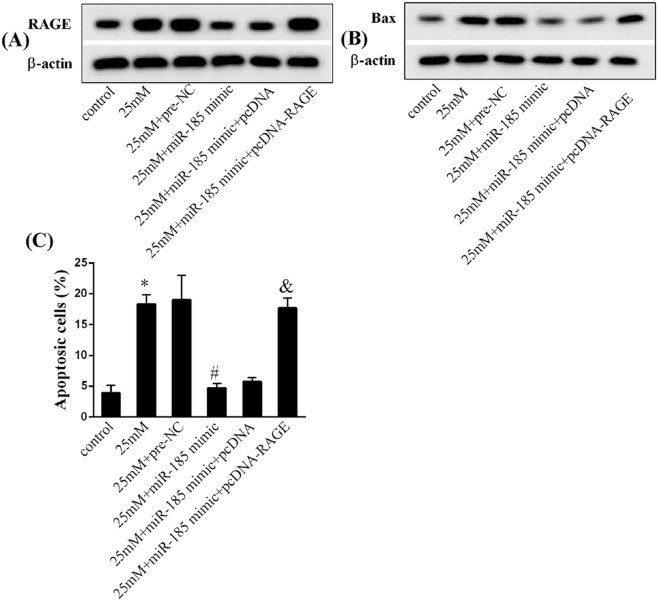
Effect of miR-185 overexpression on gene expressions and endothelial cell apoptosis (**A**) The protein expression levels of RAGE in HUVECs in the groups of control, 25 mM, 25 mM + pre-NC, 25 mM + miR-185 mimic, 25 mM + miR-185 mimic + pcDNA, and 25 mM + miR-185 mimic + pcDNA-RAGE. (**B**) The protein expression levels of Bax in HUVECs in the groups of control, 25 mM, 25 mM + pre-NC, 25 mM + miR-185 mimic, 25 mM + miR-185 mimic + pcDNA, and 25 mM + miR-185 mimic + pcDNA-RAGE. (**C**) The percentage of apoptotic HUVECs in the groups of control, 25 mM, 25 mM + pre-NC, 25 mM + miR-185 mimic, 25 mM + miR-185 mimic + pcDNA, and 25 mM + miR-185 mimic + pcDNA-RAGE. Data were shown as mean ± S.D. *Compared with control group, *P*<0.05; ^#^compared with 25 mM + pre-NC group, *P*<0.05; ^&^compared with 25 mM + miR-185 mimic + pcDNA group, *P*<0.05.

Western blot analysis and flow cytometry for apoptosis detection were performed to test whether miR-185 prevents endothelial dysfunction via mediating RAGE expression. As depicted in Figure 4, exposure to high-glucose medium could facilitate the protein levels of RAGE (Figure 4A, *P*<0.05) and Bax (Figure 4B, *P*<0.05), accompanied by the enhancement of apoptotic cells (Figure 4C, *P*<0.05); however, miR-185 overexpression led to an opposite effect. Conversely, co-expression of miR-185 and RAGE restored the expressions of RAGE and Bax and promoted HUVECs apoptosis. Therefore, our results confirmed that miR-185 overexpression inhibited endothelial cell apoptosis through regulating RAGE.

### Huayu Tongmai Granules suppressed endothelial cell apoptosis via regulating miR-185/RAGE axis

**Figure 5:**
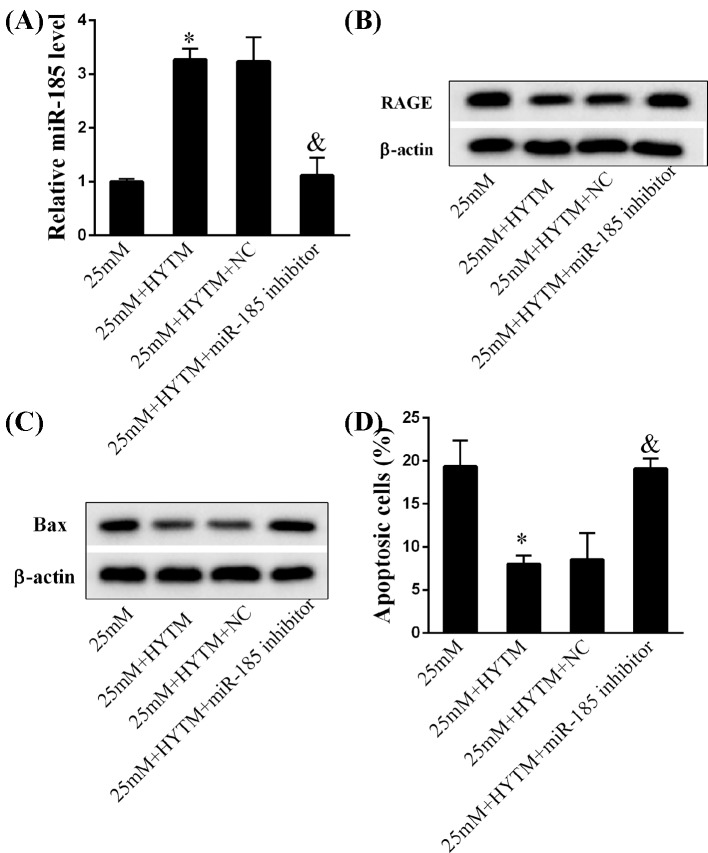
The molecular biological roles of Huayu Tongmai Granules in diabetic angiopathy (**A**) The expression levels of miR-185 in HUVECs in the groups of 25 mM, 25 mM + HYTM, 25 mM + HYTM + NC, and 25 mM + HYTM + miR-185 inhibitor. (**B**) The protein expression levels of RAGE in HUVECs in the groups of 25 mM, 25 mM + HYTM, 25 mM + HYTM + NC, and 25 mM + HYTM + miR-185 inhibitor. (**C**) The protein expression levels of Bax in HUVECs in the groups of 25 mM, 25 mM + HYTM, 25 mM + HYTM + NC, and 25 mM + HYTM + miR-185 inhibitor. (**D**) The percentage of apoptotic HUVECs in the groups of 25 mM, 25 mM + HYTM, 25 mM + HYTM + NC, and 25 mM + HYTM + miR-185 inhibitor. Data were shown as mean ± S.D. *Compared with 25 mM group, *P*<0.05; ^&^compared with 25 mM + HYTM + NC group, *P*<0.05.

To further reveal if Huayu Tongmai Granules affected miR-185 expression to inhibit endothelial cell apoptosis. The miR-185 inhibitor was used to silence miR-185 expression. As expected, miR-185 was down-regulated by the pretreatment of Huayu Tongmai Granules, while miR-185 knockdown decreased miR-185 mRNA expression (Figure 5A, *P*<0.05) under high-glucose condition. In addition, RAGE (Figure 5B, *P*<0.05) and Bax (Figure 5C, *P*<0.05) expressions at the protein levels were reduced and the apoptosis of HUVECs (Figure 5D, *P*<0.05) was deteriorated by Huayu Tongmai Granules treatment, while miR-185 knockdown resulted in an opposite trend. Taken together, our study mainly showed that 12-week Huayu Tongmai Granules treatment significantly decreased RAGE levels via up-regulating miR-185 in diabetic rats to inhibit endothelial cell apoptosis.

## Discussion

In the present study, we elucidated the beneficial effects of Huayu Tongmai Granules, a TCM for diabetic angiopathy through the establishment of a DM model in rats. We found that treatment with Huayu Tongmai Granules alleviated the blood glucose and serum ROS levels, and elevated serum NO concentration. Furthermore, its potential effects are probably mediated by the miR-185/RAGE pathway. DM and its complications are characterized by high-blood glucose levels, causing a good deal of health and social problems worldwide. Increased levels of ROS may disrupt insulin signaling and result in insulin resistance [14]. Moreover, pathogenic actions of NO are responsible to a large extent for development of complications of DM [15]. Accordingly, any medicine that can safely suppress the above symptoms would be an ideal candidate for DM therapy, and the TCM also plays an important role [16]. However, not all DM patients are suitable for some Western medicine [17]. TCM can be used as a dietary herbal supplement for DM treatment in order to decrease the side effects of some Western medicine [18, 19]. Previous studies also showed that several miRNAs are closely associated with the occurrence and development of DM and they promote or inhibit diabetic angiopathy by regulating target gene expression. For instance, Dai et al. [20] found that miR-216b could enhance angiogenesis in diabetic angiopathy through down-regulating frizzled class receptor 5. However, there is no detailed report to date on the regulation of miRNAs by Huayu Tongmai Granules. A previous study has clearly indicated that miR-185 was significantly down-regulated in diabetic patients and mice [21], and its low level was negatively correlated to blood glucose concentration [11]. MiR-185 overexpression enhanced insulin secretion of pancreatic β-cells, promoted cell proliferation and inhibited apoptosis, suggesting that overexpressing miR-185 may serve a potentially promising and efficient therapeutic approach for DM [11]. To further explore the function, target, and biological mechanisms of Huayu Tongmai Granules in the regulation of DM progression based on miRNA, we used qRT-PCR to analyze the reported miRNAs expression changes from control, DM, DM + NS and DM + HYTM group, and found that expression of miR-185 in DM group was markedly down-regulated as compared with other miRNAs, while Huayu Tongmai Granules led to an increased expression of miR-185. The results of protein expression of RAGE and Bax and cell apoptosis confirmed miR-185 as the core miRNA from Huayu Tongmai Granules in DM; Huayu Tongmai Granules decrease expression of RAGE and Bax, and so forth, inhibited apoptosis of endothelial cells.

Previous studies exist with regard to the importance of miR-185 in its regulation of cancer progression, especially in tumor cell apoptosis, such as ovarian cancers, gastric cancer, and so on [22,23]. Our study thus explored the role of miR-185 in regulating endothelial cell apoptosis, and showed an anti-apoptotic effect of miR-185 *in vitro* under high-glucose condition. The miRNAs function through inhibiting target gene expressions [24]. We further discussed the mechanism of the regulation on miR-185 after the intervention from Huayu Tongmai Granules. A previous study showed that miR-185 was a direct target of RAGE to act as a suppressor tumor in esophageal squamous cell carcinoma [10]. RAGE, an important regulator of endothelial cell growth, which is usually induced to up-regulate by hypoxia and ischemia, inhibits endothelial cell proliferation [25]. We tested endothelial cell apoptosis-related molecules RAGE and Bax with Western blot. Results showed significant down-regulation from the protein expressions of the abovementioned molecules induced by miR-185 overexpression. Further *in vitro* investigation corroborated the anti-apoptotic effect of Huayu Tongmai Granules in endothelial cells mediated by miR-185/RAGE pathway.

In conclusion, the present study suggests that Huayu Tongmai Granules showed protective role on diabetic angiopathy by elevating miR-185 expression and further down-regulating RAGE in endothelial cells.

## Supporting information

**supplementary Figure 1:** 

## Abbreviations

AGE: advanced glycation end products
DM: diabetes mellitus
ECM: extracellular matrix
HUVEC: human umbilical vein endothelial cell
HYTM: Huayu Tongmai
NO: nitric oxide
NS: normal saline
RAGE: receptor of advanced glycation end products
ROS: reactive oxygen species
TCM: traditional Chinese medicine
TBST: tris-buffered saline tween
TUNEL: terminal dUTP nick-end labeling
