# Supplementary figures and images for: Huayu Tongmai Granules protects against vascular endothelial dysfunction via up-regulating miR-185 and down-regulating RAGE
Source: Biosci Rep. 2018 Nov 30;38(6):BSR20180674. doi: 10.1042/BSR20180674 (PMC6265614; doi:10.1042/BSR20180674)

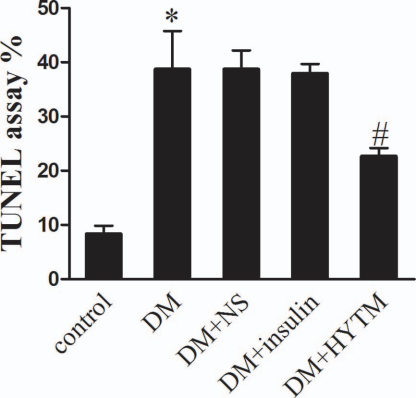

Supplement: Supplementary file 1 [file bsr20180674_Supp1.pdf]
